# Pregnancy outcomes and risk factors analysis in patients with systemic lupus erythematous

**DOI:** 10.1186/s12884-024-06701-x

**Published:** 2024-07-22

**Authors:** Jing Lu, Dan Xu, Qianqian Wan, Huijun Chen

**Affiliations:** 1 Department of Obstetrics and Gynecology, Zhongnan Hospital of Wuhan University, Wuhan, 430071 China; 2 Clinical Medicine Research Centre of Prenatal Diagnosis and Birth Health in Hubei Province, Wuhan, 430071 China; 3 Department of Rheumatology and Immunology, Zhongnan Hospital of Wuhan University, Wuhan, 430071 China

**Keywords:** Adverse pregnancy outcomes, Risk factors, Systemic lupus erythematosus, Disease activity, Lupus nephritis, Hypocomplementemia, Planned pregnancy

## Abstract

**Background:**

The management of systemic lupus erythematosus (SLE) during pregnancy remains a challenge currently. Identifying early predictors of adverse pregnancy outcomes in SLE patients can help to develop treatment plan and improve prognosis. The aim of this study is to explore the clinical and laboratory variables in the early pregnancy that can predict adverse neonatal and maternal outcomes, thereby facilitating the grading management of SLE.

**Methods:**

A retrospective analysis was conducted on 126 pregnant women with SLE who were admitted to Zhongnan Hospital of Wuhan University between January 2017 and December 2022. All enrolled patients were diagnosed (including newly diagnosed and previously diagnosed) during first trimester of pregnancy and their clinical records, laboratory results and pregnancy outcomes were reviewed. The association between the clinical and laboratory characteristics of patients at 12 gestational age and the adverse neonatal (ANOs) as well as maternal outcomes (AMOs) were analyzed.

**Results:**

A total of 117 live births (92.8%) were recorded in the study. ANOs occurred in 59 (46.8%) cases, including fetal loss in 9 cases (7.1%), preterm birth in 40 cases (31.7%), small for gestational (SGA) in 15 cases (11.9%), and complete heart block in 2 cases (1.5%). Univariate analysis showed that disease activity index (*P* < 0.0001), lupus nephritis (*P* = 0.0195), anti-SSB positivity (*P* = 0.0074) and hypocomplementemia (*P* = 0.0466) were related to ANOs. However, multivariate analysis showed that only disease activity during early pregnancy was an independent predictor for ANOs (OR = 7.053, 95% CI: 1.882 to 26.291, *P* = 0.004). In addition, 48 patients experienced AMOs during subsequent trimester, including 24 (19.0%) patients with disease flare and 23 (18.3%) patients with pre-eclampsia. Unplanned pregnancy (*P* = 0.010), active disease (*P* = 0.0004), new onset SLE (*P* = 0.0044) and lupus nephritis (*P* = 0.0009) were associated with AMOs in univariate analysis, while disease activity was identified as an independent risk factor for AMOs (OR = 2.553, 95% CI: 1.012–6.440, *P* = 0.047).

**Conclusion:**

Active disease in early pregnancy is associated with adverse pregnancy outcomes. For patients with high risk factor for ANOs and AMOs, more intensive treatment and follow-up should be a wise measure. Especially for those who suffer from active disease, they should be fully informed and given the option to terminate or continue their pregnancy.

## Background

Systemic lupus erythematosus (SLE) is a common autoimmune disease, and often occurs in women of childbearing age. With the improvement of treatment strategies and multidisciplinary management, pregnancy in patients with SLE has received unprecedented attention. As is well known, the clinical features of SLE vary among regions. Epidemiological studies have revealed that the incidence rates SLE in Asians is about 2–3 times higher than that in Caucasians [1]. Moreover, Asian SLE patients have more severe clinical features and higher positive proportion of autoantibodies than non-Asian SLE patients [2]. Therefore, the pregnancy outcomes of SLE patients in China deserve more concern.

Compared with the general population, patients with SLE are more likely to have adverse fetal and maternal outcomes during pregnancy. The incidence of miscarriage, stillbirth, preterm delivery, small for gestational age (SGA), and neonatal death has been reported to be increased in pregnancies with SLE [3–7]. In addition, the risks of SLE flare, preeclampsia, and irreversible organ damage are also increased among SLE patients during pregnancy, which contribute to maternal mortality [3–7]. In view of this, patients with SLE were commonly advised to consider pregnancy only in remission period. However, part of patients did not know they had SLE before pregnancy. They were found accidentally through prenatal examination and finally diagnosed. Moreover, even patients diagnosed before pregnancy may not be in remission at conception due to the lack of pre-conceptional counseling. For these patients, the decision to continue or terminate pregnancy should be made with caution, especially when they have passed first trimester. Thus, effective predictors that can indicate adverse outcomes as early as possible, may help us make clinical decisions and determine whether more rigorous monitoring and intervention are needed. Previous studies have identified active disease, prior nephritis, hypocomplementemia, and presence of anti-double-stranded DNA (dsDNA) antibodies as risk factors for adverse pregnancy outcomes in SLE patients [8–10]. However, the results remain controversial, and few studies have focused on the validity of predictors during early pregnancy. If the predictors can only indicate impending adverse outcomes, they will do little in improving prognosis.

We therefore conducted a single center retrospective study based on a large number of patients. The objectives of this study were to: (1) describe the frequency of adverse neonatal outcomes (ANOs) and adverse maternal outcomes (AMOs) in Chinese patients with SLE; (2) explore clinical and laboratory variables that can predict ANOs and AMOs of SLE pregnancy in early pregnancy. Our study will help to identify patients who need to strengthen management, and therefore improve the outcomes of pregnancies with SLE.

## Methods

### Study design

We retrospectively reviewed and analyzed the medical records of pregnant women with SLE in Zhongnan Hospital of Wuhan University from January 2017 to December 2022. All patients fulfilled criteria of 2019 EULAR / ACR for SLE [11], and diagnosed before 12 weeks of gestational age. Patients with incomplete medical records or experienced abortion in the first trimester of pregnancy were excluded. The study was reviewed and approved by Medical Ethical Committee of the Zhongnan Hospital of Hospital of Wuhan University (protocol code: 2,023,146 K). Verbal informed consent was obtained from all patients by telephone connection.

### Definitions

SLE activity was assessed according to SLEDAI score 2000, in which less than 4 points was considered as remission, while equal to or more than 4 points was considered as active disease [12]. Neuropsychiatric SLE (NPSLE) and lupus nephritis were defined based on criteria of 2019 EULAR / ACR for SLE [11]. Disease flare of SLE was defined based on international consensus [13]. Antiphospholipid syndrome (APS) was diagnosed according to revised Sapporo criteria [14]. Planned pregnancy refers to pregnancy in minimum of six months remission of SLE. Patients diagnosed for the first-time during pregnancy are considered as new onset SLE.

### Patients

All participants were co-managed by obstetricians and rheumatologists, and evaluated at least monthly according to recommendation [15]. Physical examination, requested laboratory tests, as well as ultrasonic examinations were performed, and medications were adjusted as needed. Clinical and laboratory data was collected from each routine evaluation records. Baseline maternal information was obtained at 12 weeks of gestation, which include age, obstetric history, pregnancy plan, and SLEDAI score. In addition, whether patients had NPSLE, lupus nephritis, thrombocytopenia (PLT < 100 × 10^9^/L), chronic hypertension, or diabetes was collected, and their medication use was also recorded. Laboratory data included complete blood counts, 24-hour urinary protein, serum creatinine, complement 3 (C3), complement 4 (C4), antinuclear antibody (ANA), anti-dsDNA antibody, anti-SSA/Ro, and anti-SSB/La antibody. Antiphospholipid (aPL) antibodies tests included lupus anticoagulant, anticardiolipin antibodies (aCL), and anti-β2-glycoprotein 1 antibodies. All laboratory tests were performed using standard methods. Data of participants on 12 weeks of gestational age were collected as variables of interest to analyze their efficiency in predicting adverse outcomes.

### Maternal and neonatal outcomes

AMOs includes mortality, lupus flare, and obstetric complications (pre-eclampsia, eclampsia, and HELLP syndrome). During pregnancy, a SELENA-SLEDAI composite was used to assess lupus flare and the severity of flare was defined as suggested [16]. Moreover, new onset or worsen NPSLE, lupus nephritis, and SLE related severe thrombocytopenia (PLT < 30 × 10^9^ /L) was recorded.

ANOs was defined as follows: (1) foetal loss, including spontaneous abortion (unexpected fetal loss before 28 weeks of gestation), therapeutic abortion (termination of pregnancy before 28 weeks of gestation due to life-threatening progression of lupus or obstetric complications), stillbirth (intrauterine fetal death after 20 weeks of gestation unexplained by chromosomal abnormalities or anatomic malformation), and neonatal death (death of a live born infant whose gestational age exceeds 28 weeks within 28 days after birth); (2) premature birth (live birth between 28 and 36 + 6 weeks of gestation); (3) SGA (birth weight below the 10th percentile according to gestational week at delivery and foetal gender); (4) asphyxia neonatorum (neonates with acidosis and Apgar score of < 7 at 1 and/or 5 min after birth) [17]; and (5) Neonatal lupus or congenital heart block.

### Statistical analysis

Statistical analysis was performed using SPSS 20.0 software. Quantitative variables were described as mean ± standard deviation, and t-test or Wilcoxon rank sum test was used to compare data between two groups. Categorical variables were described as frequency and percentage, and analyzed by chi-square or Fisher exact test as appropriate. All tests were two-tailed, and *P* < 0.05 was considered statistically significant. Variables with a statistical *P*-value < 0.05 in univariate analysis were included in multivariate analysis. The odds ratio (OR) and corresponding 95% CIs were computed.

## Results

### Clinical and laboratory characteristics of pregnancies with SLE

One hundred and twenty-six pregnancies were enrolled in the study. All participants experienced one pregnancy within the study period. The clinical and laboratory characteristics are summarized in Table 1. The mean age of patients at conception was 30.2 ± 3.5 years (range, 21 ~ 42 years). There were 97 cases (77%) of primipara and 29 cases (23%) of multipara. Thirty-six patients were newly diagnosed as SLE after conception (known as “new onset SLE”), and 90 were previously diagnosed (known as “non-new onset SLE”) with a mean disease duration of 81.6 ± 46.8 months (12 ~ 240 months) before pregnancy. In patients with non-new onset SLE, there were 86 (68.3%) patients experienced preconception consultation and planned for pregnancy. When enrolled in study at 12 weeks of gestation, 66 cases (52.4%) were in remission status, and 20 cases (15.9%) had active disease (SLEDAI > 4). In addition, 74 patients had complications, the most common of which was lupus nephritis (65 cases, 51.9%), followed by chronic hypertension (12 cases, 9.5%), thrombocytopenia (20 cases, 15.9%), and cardiac involvement (2 cases, 1.5%). APS was present in 7 patients (5.6%) and anti-La in 5 cases (4.0%). At 12 weeks of gestation, antinuclear antibodies were positive in all cases (100%), anti-dsDNA in 20 cases (15.9%), anti-SS-A in 75 cases (59.5%), anti-SS-B in 23 cases (18.2%), anti-Ro antibody in 66 cases (52.4%), anti-RNP antibody in 49 cases (38.9%). Hypocomplementemia was found in 34 cases (27.0%).

**Table 1:** Association of different characteristics during pregnancy with ANOs

Characteristics at 12 weeks gestation	Total	Adverse neonatal outcomes			Pregnancy loss		
		With	Without	*P* value	With	Without	*P* value
Number	126	59	67		9	117	
**General features**							
Maternal age ≥ 35 years	12 (9.5%)	8 (13.6%)	4 (6.0%)	0.2238	0 (0.00%)	12 (10.2%)	0.5989
Planned pregnancy	86 (68.3%)	37 (62.7%)	49(73.1%)	0.2515	3 (33.3%)	83 (70.9%)	0.028
SLEDAI > 4	41 (32.5%)	30 (50.8%)	11 (16.4%)	< 0.0001	7 (77.7%)	34(29.1%)	0.005
new onset SLE	36 (28.6%)	21 (35.6%)	15 (22.4%)	0.1168	3 (33.3%)	33 (28.2%)	0.7141
APS	7 (5.6%)	3 (5.08%)	4 (5.9%)	> 0.9999	0 (0.00%)	7 (6.0%)	> 0.9999
**Maternal complication**							
Chronic Hypertension	12 (9.5%)	9 (15.2%)	3 (4.4%)	0.0651	2 (22.2%)	10(8.5%)	0.2053
lupus nephritis	65 (51.9%)	37 (62.7%)	28 (41.8%)	0.0195	7 (77.8%)	58 (49.6%)	0.1685
Thrombocytopenia	20 (15.9%)	12 (20.3%)	8 (11.9%)	0.2282	2 (22.2%)	18 (15.4%)	0.6337
**Laboratory data**							
Anti-dsDNA positivity	20 (15.9%)	9 (15.3%)	11 (16.4%)	> 0.9999	2 (22.2%)	18 (15.4%)	0.6337
Anti-SSA positivity	75 (59.58%)	36 (61.0%)	39 (58.2%)	0.8559	5 (55.6%)	70 (59.8%)	> 0.9999
Anti-SSB positivity	23 (18.3%)	4 (67.8%)	17 (25.4%)	0.0074	1 (11.1%)	22 (18.8%)	> 0.9999
Anti-Ro positivity	66 (52.4%)	32 (54.2%)	34 (50.7%)	0.7237	4 (44.4%)	62 (53.0%)	0.7353
Anti-RNP positivity	49 (38.9%)	27 (45.8%)	22 (32.8%)	0.1478	7 (77.8%)	42 (35.9%)	0.0274
Hypocomplementemia	34 (30.0%)	21 (35.6%)	13 (19.4%)	0.0466	3 (33.3%)	31 (26.5%)	0.7015
**Medication** **During pregnancy**							
Asprin	38 (30.1%)	16 (27.1%)	22 (32.8%)	0.5613	1 (11.1%)	38 (32.5%)	0.2722
LMWH and Asprin	27 (21.47%)	12 (20.3%)	15 (22.3%)	0.8303	0 (0%)	27 (23.0%)	0.2029
Hydroxychloroquine	84 (66.7%)	32 (54.2%)	52 (77.6%)	0.007	5 (66.7%)	79 (70.1%)	0.4798

### Correlation between characteristics of SLE patients in early pregnancy and ANOs

A total of 117 live births (92.8%) were recorded in the study. The mean gestational age of live births was 36.6 ± 0.9 weeks (range 28.6–40.6 weeks). Sixty-seven cases (53.2%) achieved full-term birth without asphyxia. ANOs occurred in 59 (46.8%) cases, of which 11 cases (8.7%) had more than one ANOs (Table 2). Premature births were the most common ANOs. Fetal loss occurred in 9 cases (7.1%), including stillbirth in 4 cases (3.17%) and therapeutic abortion in 5 cases (3.97%) due to maternal or fetal complications. No spontaneous abortion or neonatal death occurred. Among 117 live births, 40 (31.7%) were premature, including 33 (26.2%) iatrogenic premature and 7 (5.56%) spontaneous premature. The rates of preterm delivery before and after 34 weeks were 15.1% and 16.7%, respectively. There were 15 cases (11.9%) of SGA recorded. There were two infants whose mother were positive for anti-SSA antibody were diagnosed with complete heart block.

**Table 2:** Fetal outcomes in pregnant women with SLE

Neonatal Outcomes	Number	Percent (%)
Without ANOs	67	53.2
ANOs	59	46.8
**Outcomes**		
Premature birth	40	31.7
Iatrogenic premature	33	26.2
Spontaneous premature	7	5.56
Delivery < 34 weeks	19	15.1
34 ≤ delivery <37 weeks	21	16.7
SGA	15	11.9
Neonatal congenital heart block	2	1.6
Asphyxia neonatorum	2	1.6
**Pregnancy loss**	9	7.1
Spontaneous abortion	0	0.7
Therapeutic abortion	5	3.97
Stillbirth	4	3.17
Neonatal death	0	0

To explore risk factors that can predict adverse outcomes at early stage of pregnancy, we analyzed the correlation between the clinical and laboratory characteristics of SLE patients at 12 weeks gestation and ANOs (Table 1). Univariate analysis revealed that the presence of lupus nephritis, active disease, positive anti-SSB, and hypocomplementemia in early pregnancy were associated with ANOs (*P* < 0.05). For the patients with thrombocytopenia, the probability for ANOs was increased but showed no significant (*P* = 0.228). In multivariate analysis, only SLE activity (OR = 7.053, 95% CI: 1.882 to 26.291, *P* = 0.004) was independent predictor for subsequent ANOs (Table 3). Predictors for fetal loss is summarized in Table 1. Univariate analysis showed that cases with active disease, unplanned pregnancy, or anti-RNP positivity tended to experience pregnancy loss (*P =* 0.005, *P =* 0.028, *P =* 0.027, respectively). However, none of them was independent predictor for fetal loss in multivariable analysis.

**Table 3:** Multivariable analysis of different characteristics during early pregnancy with ANOs

Characteristics at 12 weeks gestation	Total	Adverse neonatal outcomes			multivariable analysis		
		With	Without	*P* value	OR	95% CI	*P* value
Number	126	59	67				
Planned pregnancy	86 (68.3%)	37 (62.7%)	49(73.1%)	0.2515	1.040	0.146–7.410	0.968
SLEDAI > 4	41 (32.5%)	30 (50.8%)	11 (16.4%)	< 0.0001	7.053	**1.882–26.291**	**0.004**
new onset SLE	36 (28.6%)	21 (35.6%)	15 (22.4%)	0.1168	2.035	0.284–14.563	0.479
lupus nephritis	65 (51.9%)	37 (62.7%)	28 (41.8%)	0.0195	0.599	0.180–1.988	0.402
Hypocomplementemia	34 (30.0%)	21 (35.6%)	13 (19.4%)	0.0466	3.229	0.694–15.018	0.135
Hydroxychloroquine	84 (66.7%)	32 (54.2%)	52 (77.6%)	0.007	0.821	0.291–2.316	0.709

### Correlation between new onset SLE/planned pregnancy and ANOs

The mean SLEDAI score of new onset SLE patients was 6.0 ± 5.4 at 12 weeks of gestation, and 18 cases (50.0%) were in active status at the time of enrollment. Among new onset SLE cases, 21 cases (58.3%) developed ANOs, including 3 cases of pregnancy loss, 16 cases of preterm birth, and 7 cases of SGA. The mean SLEDAI score of 90 non-new onset patients was 3.0 ± 3.2 at 12 weeks of gestation, and 23 cases (25.6%) had active disease during first trimester of pregnancy. In non-new onset SLE cases, 38 ANOs (42.2%) occurred during pregnancy (6 with pregnancy loss, 24 with preterm birth, and 8 with SGA), and up to 70.0% ANOs occurred in cases with active disease in early pregnancy. It is worth noting that the ANOs rate of new onset cases was higher than that of non-new onset cases but showed no significant (Table 1, *P* = 0.1168), and the pregnancy loss rate was also similar between these two groups. In addition, for the planned pregnant women, the rate of ANOs and pregnancy loss were significantly lower when compared with unplanned pregnant women, but only significant difference was observed for the latter (*P* = 0.2515 and *P* = 0.028).

### Correlation between medicine application in early pregnancy and ANOs

As for treatment, 55 cases (43.7%) used prednisone (mean dose11.6 ± 9.5 mg/d), 31 cases (24.6%) used meprednisone (the average dose 9. 1 ± 4.5 mg/d) and 24 cases (19%) combined with immunosuppressive medications (azathioprine, tacrolimus or cyclosporine A) during the first trimester of pregnancy. Eight-three (65.9%) patients with non-new onset SLE continued their previous medicine regime at conception, and 19 patients with new onset SLE began to use glucocorticoids after diagnose. In addition, 84 cases (66.7%) took hydroxychloroquine, 38 cases (30.1%) received aspirin alone and 27 cases (21.47%) were treated with aspirin and low molecular weight heparin. Among patients without glucocorticoids use at the time of enrollment, 12 new onset ones and 12 non-new onset ones required medicine initiation in subsequent trimester. Univariate analysis revealed that patients who used prednisone more than 15 mg/d or combined with immunosuppressants in early pregnancy were more likely to have ANOs. However, the high rate of ANOs may also be caused by the disease itself. For the patients treated with high dose of drugs, half of them were non-planned pregnancy or new onset SLE. Moreover, univariate analysis revealed that hydroxychloroquine application was associated with reduced risk of ANOs (*P* = 0.0071).

### Correlation between characteristics of SLE patients in early pregnancy and AMOs

Clinical features of AMOs during pregnancy were shown in Table 4. Ninety-four patients were in remission, 30 patients were in mild to moderate activity and only 2 patients in severe activity when they were enrolled in study. Their mean SLEDAI score was 6.7 ± 4.4 at 12 weeks of gestation. This may be because patients with severe SLE activity in early pregnancy tend to choose to terminate pregnancy. There were no maternal deaths. Of all patients, 48 (38.1%) experienced AMOs during subsequent pregnancy. Lupus flare occurred in 24 patients (7.9% in the second trimester, and 11.1% in the third trimester), and 14 (11.1%) patients had severe flares. Among women who flared, two-third had renal involvement, 2 patients (8.3%) had skin manifestations, one patient (4.2%) had articular symptoms, and 11 patients (45.8%) had hypocomplementemia. Three patients experienced multiple organ involvement, including pulmonary hypertension, kidney damage, central nervous system and blood system involvement. All patients with disease flare required for intensified treatmentcation (e.g., an increase of steroid dosage or addition of immunosuppressants) in later pregnancy. In addition, preeclampsia occurred in 23 (18.3%) patients, and no eclampsia or HELLP syndrome was observed. Since it is difficult to completely distinguish flare and eclampsia in some patients, we consider both as AMOs for statistical analysis. It was showed that unplanned pregnancy, lupus nephritis, new onset SLE, and SLE activity were associated with AMOs (Table 4, *P* = 0.01, *P* = 0.0009, *P* = 0.0044, *P* = 0.0004). Multivariable analysis indicated disease activity was identified as the independent risk factor for AMOs (OR = 2.553, 95% CI: 1.012–6.440, *P* = 0.047) (Table 5).

**Table 4:** Association of different characteristics during pregnancy with AMOs

Characteristics at 12 weeks gestation	Total	Adverse maternal outcomes		*P* value
		With	Without	
Number	126	48	78	
**General features**				
Maternal age ≥ 35years	12 (9.5%)	7 (14.6%)	5 (6.4%)	0.2094
Planned pregnancy	86 (68.3%)	26 (54.1%)	60 (76.9%)	0.010
SLEDAI > 4	41 (32.5%)	25 (52.0%)	16 (20.5%)	0.0004
new onset SLE	36 (28.6%)	21 (43.7%)	15 (19.2%)	0.0044
APS	7 (5.6%)	1 (2.1%)	6 (7.7%)	0.2504
**Maternal complication**				
Chronic Hypertension	12 (9.5%)	8 (16.6%)	4 (5.1%)	0.0574
lupus nephritis	65 (51.9%)	34 (70.8%)	31 (39.7%)	0.0009
Thrombocytopenia	20 (15.9%)	6 (12.5%)	14 (17.9%)	0.4631
**Laboratory data**				
Anti-dsDNA positivity	20 (15.9%)	9 (18.8%)	11 (14.1%)	0.6165
Anti-SSA positivity	75 (59.58%)	28 (58.3%)	47(60.2%)	0.8536
Anti- SSB positivity	23 (18.3%)	8 (16.7%)	15 (19.2%)	0.8147
Anti-Ro positivity	66 (52.4%)	26 (54.2%)	40 (51.3%)	0.8546
Anti-RNP positivity	49 (38.9%)	17 (35.4%)	32 (40.2%)	0.5761
Hypocomplementemia	34 (30.0%)	13 (27.1%)	21 (26.9%)	> 0.9999
**Medication During pregnancy**				
Asprin	38 (30.1%)	16 (33.3%)	23 (29.5%)	0.6940
LMWH and Asprin	27 (21.47%)	11 (22.9%)	16 (20.5%)	0.2335
Hydroxychloroquine	84 (66.7%)	31 (64.6%)	53 (67.9%)	0.6965

**Table 5:** Multivariable analysis of different characteristics during early pregnancy with AMOs

Characteristics at 12 weeks gestation	Total	Adverse maternal outcomes			multivariable analysis		
		With	Without	*P* value	OR	95% CI	*P* value
Number	126	48	78				
Planned pregnancy	86 (68.3%)	26 (54.1%)	60 (76.9%)	0.010	1.040	0.146–7.410	0.968
SLEDAI > 4	41 (32.5%)	25 (52.0%)	16 (20.5%)	0.0004	2.553	**1.012–6.440**	**0.047**
new onset SLE	36 (28.6%)	21 (43.7%)	15 (19.2%)	0.0044	1.767	0.362–8.615	0.481
lupus nephritis	65 (51.9%)	34 (70.8%)	31 (39.7%)	0.0009	1.801	0.746–4.348	0.191

## Discussion

SLE remains a high-risk situation with higher adverse maternal and fetal outcomes. The earlier the high-risk group is identified, the more beneficial it is for clinical decision-making and grading management [18]. Therefore, our study analyzed the correlation between clinical and laboratory characteristics of SLE patients at 12 gestational weeks and their pregnancy outcomes to determine risk factors that can predict subsequent pregnancy outcomes. It was found that disease activity, lupus nephritis, anti SSB and hypocomplementemia were associated with ANOs, while unplanned pregnancy, lupus nephritis, new onset SLE and active disease were associated with AMOs. It indicates that patients with these factors deserve more intensive treatment and follow-up during pregnancy. More importantly, as disease activity is an independent risk factor for ANOs and AMOs, even patients with mild or moderate activity should be fully informed and a decision on whether to continue pregnancy should be cautiously made.

Regarding the fetal/neonatal outcomes, previous report indicates that patients with SLE have up to 22% of pregnancy losses, including early miscarriage and fetal death. Our research showed that the proportion of ANOs in SLE patients was as high as 46.8%, with fetal loss accounting for 7.1% and preterm birth accounting for 31.7%. However, early spontaneous miscarriage was rare in this study. This may be because the patients had already passed early pregnancy at the time of enrollment and timely diagnosis and treatment was given. In addition, SLE combined with APS, thrombocytopenia, proteinuria, and chronic hypertension is associated with an increased risk of miscarriage, low birth weight infants, and premature birth [19]. The laboratory characteristics, such as anti dsDNA, are considered as indicators of disease activity level and poor prognostic of SLE [20], and anti SSA/SSB antibodies are verified to be the cause of fetal atrioventricular block. However, little study focused the value of indicators for ANOs at early stage of pregnancy, which may contribute to the graded management of SLE patients. Our study found a correlation between SLE disease activity, lupus nephritis, anti-SSB as well as hypocomplementemia and ANOs, suggesting that patients with these risk factors should be given more attention and intensive treatment under multidisciplinary management during pregnancy. Anti- SSB and hypocomplementemia may be a new indicator for ANOs, but this needs further confirmation. More importantly, multivariate analysis shows that only disease activity is an independent risk factor for ANOs. At present, guidelines recommend terminating pregnancy only for SLE patients with obvious disease activity in early pregnancy. We thought that even patients with mild or moderate disease activity should also be fully informed and given the option to terminate or continue their pregnancy, especially for those with poor response for treatment in short period.

Some methods have been attempted to improve pregnancy outcomes in SLE, and planned pregnancy is considered a promising one [21]. Our results indicate that although planned pregnancy did not have a significant impact on ANOs, it significantly reduces the risk of pregnancy loss. The importance of planned pregnancy is worth more efforts of rheumatologists and obstetricians. Due to some patients not knowing they have SLE before pregnancy, it is difficult to completely avoid new onset cases. Previous study reported that there was no significant difference in ANOs between new onset patients and non-new onset ones [22]. Similar results were also observed in our study. This further indicated that disease activity rather than new onset was the factor affecting ANOs, and timely treatment is crucial for improving prognosis. As for medicines, only hydroxychloroquine application was associated with reduced risk of ANOs. It was reported that sustained use of hydroxychloroquine could significantly reduce the risk of CHB and neonatal lupus syndromes. For here, we found the benefits of hydroxychloroquine in improving other fetal outcomes. The positive effects of aspirin and low molecular weight heparin have not been observed in our study, which may be due to the limited number of APS patients.

Regarding AMOs of SLE, two issues received great concern, namely disease flare and concurrent preeclampsia, both of which seriously threaten maternal health. Currently, it is agreed that pregnancy may lead to higher rates of disease flares, widely variable flare rates of between 25 and 65% [23–27]. Renal and hematologic flares are common. Possible high-risk factors for “flare” include first pregnancy [28, 29], multiple exacerbation before pregnancy [27], active disease during the 6 months prior to conception, history of lupus nephritis, and positive serum anti ds DNA antibodies at conception [30–32]. Another issue is pre-eclampsia, complicating 16–30% of SLE pregnancies. Its incidence risk is 3–5 times higher in SLE patients compared to general population [33]. In our study, although all patients received treatment from early pregnancy onwards, 38.1% of patients experienced AMOs, including 24 cases with disease flare and 23 cases with preeclampsia. Unplanned pregnancy, lupus nephritis, new onset SLE, and SLE activity were associated with AMOs, which is in accordance with previous reports. It suggested that for the patients with impaired target organ function or lack of systematic management before pregnancy, more intensive treatments should be considered to avoid disease exacerbation as pregnancy progresses. In addition, multivariable analysis indicated disease activity as the independent prognostic factor for AMOs, suggesting a fully evaluation should be performed before conception in these patients.

This study is limited by the small sample size and retrospective method. Several considerations should be considered when interpreting the findings. First, all enrolled patients have passed the first trimester, thus the impact of diseases on early pregnancy was not evaluated. Secondly, all patients received treatment from early pregnancy, and those without multidisciplinary management may have a poor prognosis. Finally, the data was from one single center, so the generalization of findings is limited. Large-sample and multicenter studies are necessary to further elucidate the value of predictors mentioned above.

## Conclusion

Although improvements in treatment have enabled majority of SLE patients have opportunity of pregnancy, improving adverse pregnancy outcomes remains a challenge for management. Careful history collection and appropriate examination can aid in early detection, while early evaluation of clinical and laboratory characteristics can help classify high risk population. We have identified factors at 12 gestational weeks that could predict ANOs and AMOs and suggest conducting graded management based on these factors. Although our study has limitations, it provides some insights into exploring early predictors for subsequent outcomes in SLE patients.

## Data Availability

All data generated or analysed during this study are included in this published article.

## Abbreviations

SLE: systemic lupus erythematosus;
SGA: small for gestational age;
dsDNA: double-stranded DNA;
APOs: adverse pregnancy outcomes;
APS: antiphospholipid syndrome;
AMOs: adverse maternal outcomes;
ANOs: adverse neonatal outcomes;
OR: odds ratio
